# 3.A. Round table: Evidence-informed decision-making in patient care, public health, and health policy: the road ahead

**DOI:** 10.1093/eurpub/ckac129.124

**Published:** 2022-10-25

**Authors:** Organised by: European Observatory on Health Systems and Policies, Chair persons: Dimitra Panteli (Belgium), Samuel Sieber (Belgium)

## Abstract

This year marks the fiftieth anniversary of Archie Cochrane's seminal report “Effectiveness and Efficiency: random reflections on health services”, which spurred a paradigm shift in basing health policy and practice on robust empirical evidence. Indeed, evidence-informed decision- and policy-making has since permeated different levels of healthcare, public health, and health systems. More recently, the notion of “evidence-based” medicine has seen a shift towards “evidence-informed” health policy and practice, explicitly acknowledging that research evidence is but one of several factors influencing decision-making. In patient care, clinical expertise and a patient's own values and beliefs influence treatment and care; while in health policy-making, political and social factors, financial concerns, timing, and stakeholder relationships are decisive to successful implementation. Nonetheless, evidence-informed decision-making in health revolves around several core tenets, including a clear and well-communicated definition of objectives, using best-available, empirical evidence sourced from multiple sources, excellence and interdisciplinarity in the methods of analysis, and active collaboration across sectors and stakeholders to drive change. Across healthcare, public health, health systems and global health, actors continue to adapt these core principles into increasingly sophisticated strategies of evidence-informed decision-making. In the shadow of the global COVID-19 pandemic, exchanging best practices across these areas of application within the complex evidence ecosystem is a key driver for building more sustainable capacities in evidence-informed decision-making and partnerships to accelerate progress towards strengthening health systems and achieving global health equity. This panel aims to bring together major actors in knowledge translation and evidence-to-policy processes, highlighting latest resources and their relevance to public health, and stimulating collaboration and future action. With brief inputs from knowledge brokers, such as the European Observatory on Health Systems and Policies and the World Health Organization's (WHO) Evidence-informed Policy Network (EVIPNet), the panel will reflect on the current state of evidence-informed policy- and decision-making, showcase latest tools and resources, and discuss them from the specific perspectives of public health, coverage (with a focus on Health Technology Assessment), and patient and citizen involvement (with a focus on shared decision-making). Contributions from the audience will be sought in an open discussion round following initial inputs from panelists, with the objective of further shaping collaboration with involved stakeholders towards improving population health.

**Key messages:**

• Building on current best practices in evidence-informed decision-making is crucial to achieving better outcomes in healthcare, public health and health systems.

• Leveraging synergies and collaborating between different areas of application of evidence-informed decision-making can help ensure maximum impact and strengthen the evidence ecosystem.

**Speakers/Panellists:**

**Suszy Lessof**

European Observatory On Health Systems and Policies, Brussels, Belgium

**Tanja Kuchenmueller**

WHO, Geneva, Switzerland

**Elena Petelos**

CSFM & HSR-PH Lab, Faculty of Medicine, University of Crete, Iraklion, Greece

**Ansgar Gerhardus**

German Public Health Association - DGPH, Bremen, Germany

**Isabelle Scholl**

University Clinic Hamburg-Eppendorf, Hamburg, Germany

